# Developing Teaching Practice in Computational Thinking in Palestine

**DOI:** 10.3389/fpsyg.2022.870090

**Published:** 2022-06-10

**Authors:** Abdel Ghani, David Griffiths, Soheil Salha, Saida Affouneh, Fakher Khalili, Zuheir N. Khlaif, Daniel Burgos

**Affiliations:** ^1^Faculty of Educational Sciences, An-Najah National University, Nablus, Palestine; ^2^Research Institute for Innovation & Technology in Education (UNIR iTED), Universidad Internacional De La Rioja, Logroño, Spain

**Keywords:** in-service training, computational thinking, pedagogy, thinking steps, challenges to adoption

## Abstract

Sporadic efforts have been made to introduce computational thinking methods into K-12 education in Palestine, but these have been held back by the challenging educational environment. However, a recent in-service training initiative, funded and organized by the Ministry or Education of Palestine, constitutes a significant effort to embed computational thinking in K-12 practice. The middle school teachers who participated in the training course were invited to participate in the present study, and 38 did so. A qualitative approach involving both interviews with teachers and classroom observations was used in data collection. All the teachers agreed to be observed in their classrooms, while 20 of the 38 also agreed to participate in the interviews. The findings showed that teachers of a range of topics, including social sciences and languages, employed computational thinking skills in teaching their students, but they were confronted by a number of challenges, including technical infrastructure and support, and a lack of time to prepare CT classes and space in the curriculum to deliver them. The results indicate that the most appropriate action to support teachers’ delivery of CT would be to provide peer exchanges and expert coaching in the integration of CT in the curriculum.

## Introduction

This paper reports on the challenges experienced in Palestine in the adoption of computational thinking (CT) methods in K-12 schools, and on an intervention made by the Ministry of Education of Palestine (MoE) to address these challenges. In doing this, it provides insight into a previously invisible body of CT practice in a country living with conflict and economic insecurity, and an exploration of how the challenges of implementing CT can be addressed in such a context.

In alignment with the worldwide trend to the inclusion of CT in the curriculum, many schools and teachers in Palestine have introduced elements of computational thinking concepts and skills. This has frequently been in the absence of any official program, often through participation in national and international programming competitions. Indeed, the teachers who were responsible for this practice were often unaware that the activities that they were carrying out could be classified as CT.

Teachers, schools, and the MoE in Palestine are all keen to contribute to the development of CT skills in the classroom but are faced with two problems. Firstly, there is a lack of practical guidance on how CT can best be implemented with K-12 learners in different curricular areas. Secondly, schools, teachers, and learners vary greatly around the world, and there is a lack of studies in diverse K-12 educational contexts, making it challenging to develop appropriately focused policies, to provide effective support for teachers, or to share practice. As a result, teachers lack guidance on how to approach the teaching of CT in their own context, and this difficulty is often compounded by their misconceptions of what CT involves.

In response to these challenges, in 2019 the MoE established a training program to support and extend emerging CT practice in K-12 schools, with a focus primarily on STEM subjects and language classes, with a much smaller participation from social sciences teachers. The research reported here was carried out within the context of this intervention, taking advantage of the opportunity to collect valuable data about CT practice. It aims to provide insight into the challenges faced by teachers of CT in K-12 schools in Palestine, the support which is provided for them in designing CT strategies in their teaching, and the strategies that they have adopted in using CT approaches in the classroom.

## Literature Review

The practice of the authors in developing support for teachers in the adoption of CT approaches has been informed by ongoing engagement with the literature. In literature review, we summarize the publications which have guided our practice and research. We outline the concepts and approaches to CT which have been merged in our research framework and identify the gaps which we address in this paper.

### Approaches to Computational Thinking

The origins of Computational Thinking (CT) in primary and secondary schools have been discussed in many publications and can only be addressed briefly here. The term was introduced by Papert (1980) but became prominent following influential interventions by Wing (2006, 2011). She proposed that in higher education it would benefit everyone to learn how to think like a computer scientist and made a recommendation to “expose pre-college students to computational methods and models” (Wing, 2006, p. 35). Accordingly, she suggested that “to reading, writing, and arithmetic, we should add computational thinking to every child’s analytical ability” (Wing, 2006, p. 33). Wing (2011) defined computational thinking a “the thought processes involved in formulating problems and their solutions so that the solutions are represented in a form that can be effectively carried out by an information-processing agent.”

Wing’s proposals became increasingly prominent and were supported by the National Science Foundation in the United States, which provided substantial funding to implement the approach in schools. In Europe, similar priorities were emerging at the time of Wing’s initial paper, and the Key Competences for Lifelong Learning set out that.

Individuals should have skills to use tools to produce, present and understand complex information and the ability to access, search and use internet-based services. Individuals should also be able use IST (Information Society Technologies) to support critical thinking, creativity, and innovation (European Council, 2006, p. 1).

This concern with digital skills, and within that category computational thinking, was in large part driven by the concern that countries would be unable to compete as the digital economy unfolded, as acknowledged by the European Commission: “skills will to a great extent determine competitiveness … Many sectors are undergoing rapid technological change and digital skills are needed for all jobs” (European Commission, 2016). In support of this strategy, studies have indicated that CT activities in the classroom can increase students’ self-efficacy in both CT and STEM (Feldhausen et al., 2018; Kwon et al., 2019).

Over a number of years, work has been underway to provide evidence of the benefits of the use of CT in K-12 classrooms. For example, Israel et al. (2015, p.273) found that “struggling learners generally thrived in the computing environments,” while Hava and Ünlü (2021) have identified a correlation between the middle school students’ computational thinking skills and their STEM career interest and attitudes toward inquiry. The documentation of the benefits of CT in such studies has led to widespread initiatives to include CT in the educational process and curriculum, with a concomitant need to provide appropriate teacher training. Early in the emergence of the CT approach, Barr and Stephenson (2011) identified a lack of the knowledge and skills required to integrate CT into school curricula. More recently, the implications of CT for teacher education were reviewed by Yadav et al. (2017). Work has also been carried out to identify appropriate pedagogical approaches (Yadav et al., 2016), and studies of teacher education in CT have been carried out, for example by Hirsh and Baronak (2020) in the context of pre-service training, and by Rich et al. (2021) in relation to continuous professional development.

As described above, Wing’s original formulation of CT involves “thinking like a computer scientist,” and so, it is perhaps unsurprising that the terms CT and Computer Science (CS) are often used interchangeably in both theory and practice. However, it is necessary to distinguish them in order to provide guidance for teachers. This distinction has proved hard to finalize, and this has given rise to the many definitions in the literature. An overview of some of the definitions and approaches that have been proposed are available and Bocconi et al. (2016). An influential recent formulation is that of Denning and Tedre (2019):

Computational thinking is the mental skills and practices for:

- Designing computations that get computers to do jobs for us.
- Explaining and interpreting the world as a complex of information processes (Denning and Tedre, 2019, p. 4).

To support teachers in the task of supporting children’s learning, a many specialized computer applications has been developed, including mobile and tablet-based apps. A long-standing example is Scratch and, more recently, Scratch Jr., and the impact of the latter is reviewed by Papadakis (2021). Papadakis (2022) also comments that “Many apps offering various programming lessons, puzzles, and challenges to teach core coding concepts to children have increased in recent years,” but adds that it is not yet clear what children learn from them. Many of these apps take a ludic approach to CT, as championed in Bers’ book “Coding as Play” (Bers, 2020). An important class of applications is block-based programming environments enabling children to manipulate code through touch screens, Lin and Weintrop (2021) review work taking this approach and discuss its relationship with textual programming environments.

### Theoretical Framework

The authors have worked over a number of years to encourage school students to apply to study STEM subjects at university level, with a particular emphasis on the participation of women. In the course of this practice, conceptions of CT have been identified which are valuable in addressing the challenges which are experienced in the Palestinian context, and these have come together to constitute our research framework. We make the broad distinction that CS is a discipline that focuses on the study and development of computers and algorithmic processes, whereas CT is a broader set of strategies that can help to solve problems in ways that computers can understand. More specifically, we follow the definition provided by the ISTE (2011) which operationalizes CT as a problem-solving process that is based on:

- Formulating problems in a solvable way.
- Organizing and analyzing data logically.
- Use abstractions to represent data.
- Automating solutions through algorithmic thinking.
- Identifying, analyzing, and implementing possible solutions with the goal of achieving the most efficient and effective combination of steps and resources.
- Generalizing and transferring this problem-solving process to a wide variety of problems.

From this perspective, the integration of CT into the curriculum is differentiated from teaching the discipline of computer science, in which learners are asked to follow the detailed processes of computation, and, in effect, to think like a computer when dealing with problems in any field (Grover and Pea, 2013). Nevertheless, many computer science principles can be included among CT concepts or processes, and, as a result, it is not a simple matter to tease out the implications for teaching practice of a decision to adopt a CT approach. In our research, we identify the following thinking steps as the components or processes of CT:

- Algorithmic thinking: clearly specifying and arranging a sequence of steps to solve a problem, using mathematical, symbolic, logical, and textual expressions (Weese, 2017; Denning and Tedre, 2019, p.84).
- Abstraction: a mental activity that isolates a specific characteristic of something on a conceptual level, while discarding information (Istikomah and Budiyanto, 2018; Park, 2019). Abstraction can also involve creation of a generalized representation or model of a complex problem (Weese, 2017).
- Problem decomposition: the process of dividing a complex problem into small elements or subproblems, so that each element can be dealt with separately (Istikomah and Budiyanto, 2018; Rijke et al., 2018; Palts and Pedaste, 2020).
- Data management: tasks include collection, representation, and analysis of the quantitative or qualitative values of variables related to a thing or phenomenon. Data management are generally facts or information that are collected for reference or analysis.
- Parallelization: the “simultaneous processing of smaller tasks from a larger task to reach a common goal” (Sampson et al., 2018, p.196), which may also improve efficiency (Palts and Pedaste, 2020).
- Control flow; the process determining the completion of the steps of an algorithm (Istikomah and Budiyanto, 2018), which may repeat specific steps many times, complete steps under certain conditions, skip steps, or stop a process before all steps are finished.
- Visualization: the use of different representations, such as maps, photos, and drawings, to create and improve conceptual understanding among learners (Cetin and Andrews-Larson, 2016)

It is these thinking steps which we have sought to develop in our practice, and which we use as an analytical tool in examining the MoE summer school which is the focus of this study. Rather than seeing these thinking steps as discrete curricular items, we follow the approach recommended by Pears et al. (2019), who argue for seeing CT as an integrative element across the curriculum, rather than as a separate study. Given the practical challenges of defining the scope of CT in the classroom across the curriculum, we seek to bring simplicity and clarity to our aims and activities by providing support to teachers in developing their pedagogic skills in the three areas defined the framework set out by Brennan and Resnick (2012). “computational concepts (the concepts designers engage with as they program, such as iteration and parallelism), computational practices (the practices designers develop as they engage with the concepts, such as debugging projects or remixing others’ work), and computational perspectives (the perspectives designers form about the world around them and about themselves).” In their framework, Brennan and Resnick were building on their work with the Scratch programming environment, and we have worked extensively with this environment in our training activities.

### Gaps in Knowledge of Teacher Education and Computational Thinking

Since the emergence into prominence of CT, a great deal of research has been carried out to examine its impact on the way that students solve problems, specifically in mathematics and science subjects, for example, Yuen and Robbins (2014), Chen et al. (2017), Park (2019), and Hava and Ünlü (2021). However, Voogt et al. (2015) identified that relatively little attention had been paid to teachers’ thinking, and to teacher education in CT. In recent years work, some work has been carried out to fill this gap, with researchers starting to explore in-service and pre-service teachers’ knowledge and their practices in including CT in classrooms.

In their review of 2019, Mason and Rich found that few studies had been carried out into the education of teachers at elementary level, or their impact:

(a) few studies have been published about training elementary school teachers to teach computing, coding, and programming, with slightly more studies on preservice teacher training than in-service PD; (b) interventions have focused more on developing elementary CS teachers’ content knowledge than their pedagogical knowledge; (c) studies overwhelmingly showed that training can improve teachers’ self-efficacy, attitudes, and knowledge, even over relatively short interventions; and (d) the literature has said little about whether or to what extent changes in self-efficacy, attitudes, and knowledge lead to changes in actual practice or improved student learning (Mason and Rich, 2019, p. 809).

The need for practical studies with teachers which can shed light on how they can be best supported is echoed by Mäkitalo et al. (2019). Writing with Matti Tedre (co-author of the influential book *Computational Thinking* (Denning and Tedre, 2019) whose definition of CT we discuss above), they put matters rather more strongly: “CT literature has pointed out a dire need for more research on understanding teacher learning and how to support their learning in the best way in pre- and in-service teacher education.” They identify three problems that CT is facing, the third of which is a lack of understanding on “How do teachers learn to synthesize CT with existing content and pedagogical strategies?” Little substantial work has been carried out in this area, and in particular concerning in-service training, and the valuable studies that have been carried out, for example Kong et al. (2020), have only started to fill the gap.

The present research is a contribution toward the effort to fill these gaps in the knowledge of how to address teachers’ needs for training and support in introducing CT, how they integrate CT into their practice. We also address another gap, which is largely unidentified in the research literature: to understand the challenges of adopting CT approaches in contexts which are quite different from the wealthy and powerful countries in which the approach was first conceived. In the following section, we document and describe this context.

### The Educational Context

#### A Challenging Environment

Historically, the Palestinian education system has faced many challenges and difficulties, and for several decades the formal education has been administered and controlled by external authorities as a consequence of political instability. In 1967, Israel occupied the West Bank, including East Jerusalem, and the Gaza Strip. Israeli military authorities targeted the education system especially during the first intifada (1987–1992; Abo Hommos, 2013), including measures, such as closing down many schools and universities for more than 2 years, arresting or killing teachers or students, and imposing arbitrary measures to create poor learning environments (Ramahi, 2015). Israeli policies have had an enormous impact on Palestinian education, leading to a long-lasting decline in academic standards at all levels of education (Nicolai, 2007), and arbitrary Israeli policies have resulted in the weakening of the Palestinian education system in general. However, these Israeli policies and arbitrary measures did not entirely prevent Palestinians from preserving their cultural and historical identity. Palestinian teachers have taken it upon themselves to confront these policies; by adopting hidden curriculum, enhancing teaching methods, engaging in qualifying courses to improve their competencies, and employing informal education to create effective learning environments (Wahbeh, 2003).

After the Oslo Accords in 1993, the Palestinians became responsible for the Palestinian education system. Consequently, the MoE was established in 1994, and has sought to prepare Palestinian children for citizenship to build their state. Currently, the MoE administers about 75% of students in the West Bank, East Jerusalem, and the Gaza Strip. The United Nations Relief and Works Agency (UNRWA) administers 15%, while private institutions administer10%. The MoE has faced great difficulties and challenges in its work to rebuild and reform the Palestinian education system, including in providing schools with qualified teachers, improving the infrastructure, and designing modern educational curricula to meet the aspirations of the Palestinian people (Ramahi, 2015).

#### Teacher Education and Professional Development

In 1998, the MoE tried to standardize teacher qualifications, in order to enhance the professional capacity of the workforce. A bachelor’s degree was designate as the minimum qualification for a teacher entering the profession (Nicolai, 2007), but this minimum requirement did not help in improving the education system and the learning environment (Bivins, 2015). The demand for certain types of teachers, specifically those proficient in STEM, was higher than the supply during that time (Bivins, 2015). In response, teachers with math and science experience were often hired without any teaching background at all (Nicolai, 2007). Ministry of Education of Palestine (2008) established a developed a Teacher Education Strategy, and this plan remains the current strategic plan and goals. In accordance with this plan, the MoE has made strenuous efforts to requalify teachers, and, 2010 it allocated 4.5% of its annual budget to develop teachers’ competencies (UNESCO, 2010). It is therefore unsurprising that, again according to UNESCO (2010), traditional teaching strategies are still common and prominent in most Palestinian schools. These strategies include “teacher-centered” approaches, consisting of rote-learning, lecturing, and dictation. Moreover, Palestine has participated three times in the (TIMSS) study of international trends in mathematics and science for three times (2003, 2007, and 2011), and the results indicated that Palestine is still in the ranks of the lowest achievement countries (Afana, 2021), and these results are consistent with the rudimentary teaching strategies still used in these disciplines. Recent statistics are not available, but there is no reason to suppose that this negative situation has changed in recent years.

Recently Afana (2021) conducted a study of mathematical learning in Palestinian schools in challenging circumstances. According to this study, teaching loads are high, teachers’ teaching preparation is low, pre-service training does not exist, and in-service training is not effective, and universities’ teaching programs are not practical but theoretical. The significant decrease in students’ mathematics scores for students taught by teachers who studied mathematics education rather than pure mathematics was remarkable. At the same time the “Palestinian universities admission students for education with low final secondary exam scores, unlike medicine and engineering. Only few of them decide to study education with high scores because they like to become teachers” (Afana, 2021, p. 129).

#### Use of CT Strategies in K-12 Schools in Palestine

The historical and professional circumstances described in the previous sections have created an extremely challenging environment for pedagogic innovation, and as a result the widespread adoption CT by Palestinian teachers in their classrooms is still out of reach. It is therefore unsurprising that when we carried out a literature review of the integration of computational thinking by Palestinian teachers in their classrooms, we found no direct evidence. Nevertheless, there are indications that some progress has been made. The framework of the National curriculum asked teachers to develop thinking skills among students as observation, interpretation, and using various strategies to solve problems, and in 2019, the MoE initiated a training program in six of the 16 districts under its jurisdiction, to coordinate and extend this work. This initiative was the context for the research which is reported on in this paper.

## Research Design

### Research Questions

The present researchers, some enthusiastic teachers, and the MoE of Palestine are convinced of the importance of integrating CT in schools as a means of improving educational achievement and as a contribution to the development of the economy. Indeed, the workshops which provided the opportunity for data gathering for this study are evidence of the commitment of the MoE. However, if CT is to be more widely adopted in Palestine, it is necessary to create a picture of the current situation which is both more detailed and more accurate. At present it is not clear to what extent CT has been established in existing teaching practice, what challenges teachers face in introducing CT in their lessons or what support would help them in overcoming those challenges. This knowledge is needed in order to provide guidance to schools in introducing CT approaches and to design appropriate policies and supports. In addressing this need, we provide information from the Palestinian context which helps to fill the gap identified by Mason and Rich (2019, p. 809) cited above, who note the lack of studies of the training of elementary school teachers in CT methods, and of the results of that training in terms of teachers’ self-efficacy, attitudes, and knowledge.

In light of these needs, the following research questions were formulated:

1. How is computational thinking integrated into STEM and language classroom activities across different subject areas?
2. What types of support do teachers require to integrate computational thinking into STEM and language classroom instruction?
3. What pedagogical strategies do teachers report work well for using computational thinking approaches in teaching and learning?
4. What challenges do teachers face during the adoption of computational thinking in classrooms?

### Population and Data Collection

The study took advantage of the opportunity to collect data that was presented by six summer workshops that provided CT training for teachers from K-12 public schools, organized by the MoE in cooperation with the AlNayzek non-profit organization. One workshop was organized in each of the districts of Ramallah, Nablus, Jenin, Tulkarem, Qalqilia, and Salfit. Each workshop had two facilitators: an educational supervisor, whose duties include assessing the performance of teachers in their classrooms, and an expert in computer science with experience in education. In each workshop, teachers met for 2 days per week for 4 hours for 2 months. The workshops included an introduction to computational thinking and modeling of computing tools and gave teachers the opportunity to practice designing activities to integrate CT into classroom activities. During the training sessions, the teachers talked about their experience with CT in their classrooms, and training was carried out through a learning by design approach which was designing activities to be used in the class. Permission was obtained from the MoE to visit these sessions and talk with teachers about their experience.

The teachers who attended in the summer workshops were contacted through the documentation and communication channels of the workshop organizers and invited to participate in the study. Most of the teachers taught a STEM subject, but the participants were extended to include Arabic, English, and social sciences. All the teachers had made a commitment to integrate CT into their instruction, and all were willing to participate in the workshops. One of the teachers had participated in national competitions for technological initiatives. It was therefore expected that the teachers would have their own ideas about CT skills prior to the workshops, even their fields were not related to computer science. The population of the study was constituted by the 235 teachers attending the workshops, who were all invited to participate in the study, and data collection was carried out through the activities of the workshops, and in interviews with participants carried out in parallel with the workshops.

### Methods

The research approach taken in the research was selected to make the most of the unique opportunity for data collection offered by the training course. In a context where little is known of how teachers address CT in their practice, the highest priority was to obtain insight into teaching activities directly from teachers, in the real-life contexts in which they occurred (Yin, 2003). Quantitative methods were not seen as being appropriate to the study, because the self-selecting nature of the population precluded extrapolation to teaching practice across Palestine. Rather, the focus was on understanding in greater depth the practice which was being conducted by teachers who were committed and enthusiastic practitioners of CT teaching. The principal method used in the research was therefore one-to-one interviews, which ensured that teachers were not intimidated by the presence of colleagues or supervisors in discussing their practice. In view of the fact that the researchers were seeking information about an unknown body of practice, a semi-structured method was adopted for the interviews, to ensure that teachers had plenty of opportunity to raise the issues which they considered to be important. Thematic analysis was used to analyze, categorize, and report on themes within the data. Teachers’ introspection in interviews provides reliable evidence of the way in which teachers think about their practice and its purpose, but it may not be as reliable in understanding what actually occurs in classrooms during lessons, and the problems and successes which emerge. To address this, the interviews were supplemented with classroom observations carried out by the research team.

#### Semi-structured Interviews

The interview questions were developed to collect the data required to answer the research questions. The interviewer asked questions to explore teachers’ experience of integrating CT approaches into their teaching different topics in K-12 settings. The identification of teachers was based on the following criteria: attendance at all the summer workshops sessions; the willingness to integrate CT into their instruction; participation in national competitions in technological initiatives; and previous use of CT in their classroom activities. The selection process also took into consideration the need to cover a range of subjects, with different backgrounds, in order to provide data on CT integration in different contexts. The researchers scheduled the interviews at times and locations convenient to the participants.

In the interviews teachers were asked to talk about their journey using CT in teaching their topic, using the following questions (translated from the Arabic).

- Could you please give us your opinion of the workshops that you have attended?
- What did you learn from the workshops regarding CT?
- How would you describe your experience of enhancing CT strategies in your teaching?
- Could you please describe the classroom activities related to CT that you have implemented in your teaching?
- What were the main challenges that you faced while implementing CT in your class?
- You have been trained on different strategies for CT, how will you use them in your class?
- Could you please give some examples of how you implemented CT in your class and in your subjects?

After transcription of the audio files, the researchers sent the text files to the participants to make sure of they truly represented the intention of their answers in the interviews. The participants were invited to edit/add/delete the text as they saw fit. After receiving their transcripts, none of the teachers changed their answers, but few of them did add more information about the training sessions and their experiences.

#### Classroom Observations

Classroom observations enabled the researchers to examine the strategies used by teachers from different backgrounds to integrate CT in real classes, and the procedures they used to implement CT classroom activities. In order to protect privacy, no photographs were taken that showed the faces of teachers or students. During the classroom observations, field notes were taken describing the instruction of the teachers and the activities of the students. The researchers observed and documented the strategies and procedures used to integrate CT, identified the challenges encountered, and noted how teachers overcame those challenges. All the field notes were shared with participants and other members of the research team to conduct data analysis.

#### Ethical Considerations

As noted above, the workshops were conducted by team of two, one of whom was an educational supervisor from the MoE. The supervisors’ role is monitoring, advising, and supporting teachers in improving their performance. Moreover, the supervisors have considerable influence on the teachers’ career development, and the supervisors visit all teachers twice each year to write reports about their performance. A positive report on teaching practice and outcomes can lead to promotion for teachers who use new technologies in teaching, and to nomination for professional development in educational strategies and the use of technology. It was therefore essential that none of the supervisors knew which teachers participated in the study. The research was discussed with the teachers at breaks in the workshop session, and the consent process was also completed at that time. The teachers were able to choose a preferred date and time to conduct the interview.

#### Data Analysis Procedures

Analysis started during the process of data collection through writing field notes describing what was observed by the researcher. These were compiled and contrasted with the results of the interviews in identifying the findings. The procedures set out in Marshall and Rossman (2011) were applied in the data analysis. A researcher transcribed the audio recorded files as soon as an interview was completed. The names of the interviewees were anonymized to protect the identity of the participants. The transcribed file was cleaned, and unnecessary information deleted before sending it to the interviewees for review. After finishing all the interviews and cleaning, two researchers read the transcripts files in depth to identify the main ideas, concepts, and topics related to the research questions. A coding book was used as to guide the process of data analysis.

Two researchers conducted an exhaustive manual coding of the interview transcripts in order to identify CT concepts and skills. In this process, each researcher read through the transcripts and highlighted significant sections of text. In the analysis, we were only interested in studying CT concepts and skills used by practitioners in their work, and hence we focused on the portion of each interview where the interviewees described their current practice. The two researchers developed the themes and subthemes related to the research questions individually, then they met to discuss the themes and subthemes to achieve agreement on the final themes. Any disagreement appeared was solved by negotiation among the researchers.

## Results

The 235 teachers who attended in the CT workshops were invited to participate in the study, and of these, 38 gave their agreement. The teachers were all from different schools, from all six districts involved in the workshops. Table 1 shows the number of male and female teachers participating in the experiment according to the type of subject they are studying. It shows that the number of teachers who were observed during the experiment was (38), of which (22) were female, and (16) were male. And the number of teachers interviewed was (20) male and female, males (10) and females (10).

**Table 1:** Teaching area and gender of the participants in the study.

Teaching topic	Observations		Interviews	
	Male	Female	Male	Female
Science	2	3	1	2
Math	3	4	2	1
Technology	4	6	3	4
English language	2	4	1	2
Arabic language	3	2	2	0
Social sciences	2	3	1	1
**Total**	**16**	**22**	**10**	**10**

The observed classes covered a range of topics taught in fifth to ninth grade classes in different schools. Over half of the participating teachers were female.

Selection of the interviewees was made through the facilitators of the workshops, who were asked to suggest four teachers at each of the six training locations, from the 38 who had declared their willingness to participate in the project. The individual interviews ranged between 20 and 30 min and were carried out in the training centers. Of the 20 teachers who were interviewed, half were female. The interview recordings were transcribed manually in Arabic, and sections were only translated into English if this was required for publication.

We now discuss the analysis of the data as it relates to our four research questions.

### Research Question 1: How Was Computational Thinking Integrated Into Classroom Activities Across Different Contexts?

#### Group Work and Classroom Seating

Most of the activities we observed were group work activities (30 out 45 activities) and 15 were individual activities. We did not find a specific pedagogical approach which was associated with CT. Rather, the way that the teachers in this study used CT was conditioned by their approach to organizing instruction in a class, for example employing whole-class instruction or a student-centered approach. Consequently, the levels of explicit instruction that they used while implementing CT activities varied. Several teachers from different schools included CT as a student-centered activity to teach math concepts using group work in their classrooms. Their instruction began with giving directions and demonstrations to the whole class, followed by individual and collaborative work. Teachers explained the various CT tasks to the students, who then worked on those tasks collaboratively and individually.

Most of the teachers we observed (30 out of 38) changed student seating arrangements to facilitate classroom activities with CT. Some teachers changed the seats into a U shape, others ensured that students worked in a group and the rest used whole-class (traditional classroom) instruction.

#### Flexible Lesson Plans

In our observations we noticed that half of the teachers adjusted the levels of instruction to meet the needs of subgroups within the classroom and enhance students’ engagement in learning. They provided different activities for *beginners* (focused on collecting data), *novices* (who are more advanced, carrying out data analysis), and *multi-level*, all of which were carried out in the same classroom at the same time. This required flexibility and open-mindedness on the part of teachers, and some teachers modified the activities many times to meet students’ needs in the classroom. For example, teacher 5 said “Why not, I am flexible, it is easy for me to change to tasks based on students’ needs… The purpose is to integrate CT in my activities how and when… it depends on students’ needs” [Teacher 5]. On the other hand, some teachers did not depart from their existing plans, with eight teachers using a maintaining a pre-established linear approach, while six used branching structures, with different possible routes to resolve the problem.

#### Open-Ended Tasks

Seven teachers used open-ended tasks, rather than following the curriculum, especially in teaching programming. This strategy was not without risk for the teachers, as there was a danger that if they did not complete the curriculum plan by the end of the semester, there would be a negative impact on their prospects for promotion. The approach adopted by these teachers was to allow students to explore different computational thinking skills and apply them to their classroom activities. In the interviews, seven teachers reported that open tasks could be beneficial for students. For example, teacher 3 said that “open-ended tasks can allow students to explore new computational thinking skills and programming skills.” These types of activities commonly involved programming tools, such as Scratch, and languages, such as Kotlin in the eighth and ninth grades.

##### Examples of Activities and Computational Thinking Integration

Teachers used a range of activities to integrate CT into STEM and language instruction, including hands-on activities (ten activities), focusing on the processes and practices of CT, experimentation (12 activities), inquiry (nine activities), and Scratch and robots (14 activities). In math and science classes, nine teachers asked students to get data from Google Dataset about the weather, or statistical data about Middle East countries, and to visualize it to find patterns. Teachers started by showing how data could be found through a Google Dataset search, and how to access the Google data center. They then provided step-by-step instructions for students by introducing CT and instructions about how to download the data and transfer it into an Excel sheet to visualize it.

All the teachers in observed classrooms used CT steps defined by Yadav et al. (2016) in their instruction, as shown in Table 2.

**Table 2:** Thinking steps used by teachers in their instruction.

Thinking step	Definition	Topic
Data gathering	Collecting data from different resources (Internet, local community)	Mathematics, Science
Data analysis	Finding the patterns among data and understanding the characteristics of samples within teams	Mathematics, Science
Data representation	Organizing data in a suitable way using tables charts, and infographics	Mathematics, Science, Technology
Abstraction	Identifying and extracting relevant information from the activities to define main ideas	Mathematics, Languages, Social Sciences
Algorithm design	Using ordered consequences instructional steps for solving similar problems or for implementing an activity	Mathematics, Science, Technology
Decomposition	Breaking the problem and procedures into smaller parts to manage and solve it.	Mathematics, Science, Technology
Visualization	Using visual content as an easier way to understand and find patterns	Mathematics, Science, Languages, Social Sciences
Debug and correct errors	Finding mistakes in the steps to solve a problem/programming a task and fixing it	Mathematics, Technology

As may be seen, all of the thinking steps identified in our framework were applied in one or more of the seven mathematics classes which were observed. In the five science classes, all thinking steps were applied with the exception of *abstraction* and *debug and correct*. While it is perhaps unsurprising that debut and correct was not used beyond mathematics and technology, the absence of *abstraction* is interesting, given the importance that it has in scientific thinking. In the ten technology classes, the observed thinking steps aligned with some of the basic skills of programming: *data representation, algorithm design, decomposition, debug, and correct.* This raises a concern that CT activities in technology may be too tightly focused on programming, and it indicates that it would be valuable to focus training on other aspects of CT which are important aspects of technology in general and computing in particular: *data gathering, data analysis, data representation and visualization.* The 16 Languages and social sciences classes observed made use only of *abstraction* and *visualization* and thus had the narrowest range of activities despite being the largest number of classes observed. This indicates that teachers outside of the STEM subjects had difficulty in designing learning activities which could apply the concepts of CT in their classes and that it will be important to focus on this in future training if the aim of applying CT across the curriculum is maintained.

### Research Question 2: What Types of Support Did Teachers Require to Integrate Computational Thinking Into Classroom Instruction?

Thirty teachers in the study reported that they were encouraged by their school administration and supervisors to learn about ways of using CT in their instruction. Twenty-eight participants said that technical and instructional support was provided for teachers in their schools by the school administration and their colleagues. Interestingly, the nine teachers who expressed a need for training on designing CT activities emphasized the instructional design aspects rather than a need for technical support. Thirty-three said they had attended only two workshops on designing activities to use in instruction and that they felt a need for more.

#### Professional Development of Teachers

Thirty teachers said that they had the basic skills necessary to teach CT, but they did not know how to integrate these skills into their classroom activities. “If I had designed activities related to my field, I would use them… I know myself; I do not know how to integrate these skills into my classroom activities… If we had access to open-source activities, it would be beneficial for me” [Teacher 4]. It therefore appears that teachers need training in finding and using activities related to their teaching topics that use open-source applications.

During the period of this study, the teachers who participated received training to equip them with the knowledge and skills essential for CT integration in classrooms. Thirty-two teachers of thirty-eight of the participants in study were satisfied with the workshops and valued the opportunity to mix with others and learn from each other. Teacher 10 said “I am happy to be in this workshop. It provides me with skills and knowledge to implement activities.” Ten teachers in the interviews believed that these workshops would not be sufficient to gain information to integrate CT into instruction. This indicates that it would be valuable to have coaching available on site in school, so teachers can derive more benefits than can be achieved in centralized workshops.

#### Availability of Expertise

Twenty-seven out of thirty-eight teachers who participated in the study mentioned their need to communicate with experts in their subject area while integrating CT into classroom activities. Five teachers expressed their concerns about computational thinking in STEM classrooms, since they did not have a clear idea about how they would integrate it and had had bad experiences with it. In particular, the English language teachers complained about the lack of guidance on the integration of CT in classroom activities in their field. The summer professional development program went some way to meeting this need, and twenty-five teachers out of thirty-eight teachers recognized the importance of exchanging their skills and knowledge about CT during the workshops, and in the interviews, teachers said that they were more confident about integrating CT into their classroom instruction after the workshop.

Nevertheless, it is clear that there remains an unmet need for ongoing access to support from experts and peers. Thirteen teachers reported that they wanted to hear their colleagues’ stories of integrating CT into their teaching, and many teachers were influenced by those colleagues, who inspired them to implement CT skills. In the absence of such expert input and contact with peers, eleven teachers said that they used social media to ask for help, and sixteen teachers from different schools shared open resources to teach concepts in science on their pages on Facebook. Teacher 11 attested that “These activities were helpful for me.”

This shows that the opportunity to exchange experiences and expertise with their peers was highly valued by teachers.

#### Lack of Time

Time is a crucial factor that influences teachers’ use of CT approaches in their instruction. All the teachers mentioned that they needed to learn about CT, as well as about how it could be used in classrooms. However, they did not have enough time to prepare activities to integrate CT into their instruction, because of the time taken up by administrative tasks.

#### Support From School Principals Was Important for Teachers

An invitation to participate in the workshops constituted a recognition of teachers’ work, and an opportunity for teachers to develop their computational thinking, knowledge and skills, as well as helping teachers develop lesson plans and activities. Moreover, trainers could encourage teachers to develop activities and lessons, and enhance their confidence in integrating CT in classrooms.

School principals developed a clear vision and school statement about integrating CT into instruction and put it on their calendars during the academic year. Twenty teachers in the interviews mentioned that their school had clear visions about CT integration into instruction. School principals in five schools as reported by five teachers tried to minimize the risk of implementing CT into instruction. Overall, school administrations (principals and managerial staff) and teachers appreciated the administrative support, as well as colleagues’ support for computational thinking integration in classrooms.

### Research Question 3: What Pedagogical Strategies Do Teachers Report Work Well for Using Computational Thinking in Teaching?

The participating teachers used a number of different learning strategies to integrate CT concepts and practices into their instruction. Table 3 sets out the learning strategies that were used in classroom instruction, as observed and reported by teachers. These included gamification, teacher-centered lectures, problem-based learning, project-based learning, scaffolding, and collaborative learning.

**Table 3:** Learning strategies used in the classroom.

Learning strategy	Description
Problem-based learning	Six teachers of math and science divided the students into teams and then provided them with a task. The students explored the learning solution individually and reported their own learning conclusions and feedback to the team. Each student described their problem-solving procedures to the team
Group learning	Many teachers in different areas (science, technology, and English language) used this strategy. Following the instructions of the teacher, the students completed the tasks into two ways. In one class, five teams divided the work into subtasks and solved them individually, and then gathered the partial results into a final output. In another class three teams, the members were required to complete the task together, negotiate, and share their ideas to solve it
Project-based learning	Twenty-two teachers from different fields used this strategy. Teachers organized the integration of CT around projects. They introduced the tasks, which were in the form of questions, and asked students to collect, analyze and present data from Google. The students were engaged in design, problem-solving, and in making decisions based on the pattern in the data
Scaffolding	Technology, science, and English teachers used various forms of scaffolding, such as using teaching aids, chunking the activities, and modeling in their instruction to help students to carry out the CT activities
Teacher-centered lecture	Some teachers used this strategy at specific points to introduce CT concepts and to provide examples to demonstrate CT skills
Gamification	Many science and math teachers used the features of gamification (points, rewards, badges…) to introduce CT skills and concepts into instruction to enhance extrinsic motivation

Classroom observations showed that the teachers used a range of pedagogical strategies to teach the students the skills and concepts of CT. These strategies appeared to vary according to the subject in which the CT was embedded. For example, the science teachers focused on using collaborative activities to teach students CT concepts and skills. Table 3 shows the type of teaching strategy used by teachers in the classroom and its interpretation, as it was divided into problem-based learning, group learning, project-based learning, scaffolding, teacher-centered lecture, and gamification.

The richness of the pedagogical strategies contrasts with the need for pedagogical support expressed by teachers. In combination with the appreciation shown by teachers for the opportunity to exchange practice with peers, this indicates that the required pedagogic expertise is present in the community of teachers, but that the structures and time that would enable the sharing of this practice is not available to teachers. This indicates that the challenge is not to develop of appropriate pedagogical strategies for use by teachers, but rather to support them through peer exchanges and coaching.

### Research Question 4: What Challenges Do Teachers Face During the Adoption of Computational Thinking in Classrooms

In the responses to the questions in the interviews, all the teachers described challenges in integrating CT into instruction. We also noticed these challenges during classroom observations. Interestingly, when the teachers discussed these barriers, they described the challenges within the context of their instruction. Eight teachers describe the source of challenges from the infrastructure of the schools. Ten teachers described the source of the challenge from their individual characteristics, such as social commitments and lack of time. Table 4 summarizes the major challenges reported by teachers and observed during integration of CT activities, as well as the strategies that teachers used in overcoming those challenges. The barriers to integrating computational thinking reported in teachers’ interviews, together with those noted during classroom observations, were gathered into three categories, relating to students, to the learning environment, and to the teachers, respectively. We now discuss these.

**Table 4:** Challenges and strategies in adoption of CT.

**Challenges identified**		**Strategies implemented to overcome challenges**
Challenges to CT integration in instruction	Lack of suitable resources	Design activities/collaborate with other teachers to design/use existed activities.
	Lack of technical support	Ask colleagues for support/use social media to ask for help.
	Access to technology	Rotate students to the computer lab/library to use interactive projects/Internet.
	Large classes	Dividing students into groups
Challenges relating to teachers	Limited time	Break down/use video.
	Assessment	Use reflection papers and rubrics.
	Differentiation	Use peer mentoring/ scaffolding/collaboration.
	Lack of training	Use social media to get support.
Challenges related to students	Student not engaged	Use a variety of activities.
	Solving problems	Practice a lot/use many activities

#### Challenges Related to the School Environment

Thirty teachers described many factors that prevented them from integrating CT into STEM instruction including the lack of time, differentiation of strategies, assessment and training. Four challenges indicated by all the teachers and in the observations related to the learning environment including weaknesses in the technical infrastructure, large classes, lack of technical support, and lack of resources.

All the teachers mentioned that the lack of instructional time was a major challenge, although they were able to compensate for this factor by breaking down the CT activities into small chunks and carrying them out in different classes at different times. In addition, six teachers used freely available videos to explain the concepts, and designed activities to ensure that students focused on the video while viewing them. All the teachers mentioned that they were committed to finishing the mandated textbooks, while at the same time integrating CT into their instruction. “My supervisor came and asked about my progress in the curriculum. She did not ask about activities, her concern is whether I am following the plan to finish the curriculum on time or not, not that I am doing my best to use CT in class activities” [Teacher 7].

#### Challenges Related to Student Engagement

Most of the teachers mentioned that students did not fully engage in the activities because they were new to them. Teachers mitigated this challenge by using group work to encourage students to learn from each other, and to create mutual encouragement to complete the activities. Some teachers mentioned the lack of student engagement was due to the class size and the quality of the activities which were suggested to them for use in the classroom. “I believe students do not engage in the activities because it does not attract their attention, or they were feeling bored because of it… With time, maybe the quality of CT activities will be better and meet students’ needs” [Teacher 15]. Table 4 shows that teachers of computational thinking faced a challenge in the process of integrating various topics. They also experienced a lack of resources and of technical support, difficulties in access to technology and making it available to students, and overcrowded classes. At a professional level, teachers also felt that CT was being held back by their lack of time to enjoy the educational process, a lack of appreciation of teachers’ work by others, and the need to trade-off between the subjects required for the student to successfully complete the curriculum. Teachers also identified that their lack of experience and training was a challenge. At a pedagogic level, a challenge was the presence in classes of students who are not engaged in the educational process and are unable to follow the CT activities which they are given.

## Conclusion, Limitations, and Implications for Future Research

The present study set out to provide information on the challenges faced by teachers of CT in K-12 schools in Palestine, the support which is provided for them in designing CT strategies in their teaching, and the strategies that they have adopted in using CT approaches in the classroom. We now summarize the insight we have gained into our research questions.

### How Is Computational Thinking Integrated Into STEM Classroom and Language Activities Across Different Subject Areas?

The majority of the activities observed (30 of 45) involved group work and reorganization of the classroom space, but no particular pedagogic approach was used by teachers of CT, who tended to apply their default method of instruction. Half of the teachers demonstrated flexibility in providing different activities for groups of students with different levels of prior learning, while seven teachers exposed themselves to some risk by giving their students open-ended tasks rather than following the curriculum. The spread of activities across the different subject areas provided interesting results. All of the thinking steps identified in our framework were applied in one or more of the mathematics classes which were observed, and all but two steps were observed in science. In technology classes, however, the thinking steps were closely aligned with the procedures of computer programming, while languages and social sciences made use only of *abstraction* and *visualization,* despite being the largest group of classes observed. Thus, an important result is to highlight the need to focus in future training on supporting teachers outside the disciplines of mathematics and science in applying the whole range of CT thinking steps in their teaching.

### What Types of Support Do Teachers Require to Integrate Computational Thinking Into STEM Classroom Instruction?

It is interesting to note that the requirement for support was largely pedagogic rather than in development of technical skills. Most teachers said support was provided in the schools, but those who requested training emphasized a need for instructional design rather than technical support. They valued the opportunity to share practice with their peers in the workshops, but the need was expressed for ongoing coaching on site in school. The teachers unanimously identified a lack of time to learn about and integrate new methods as a significant barrier to adoption of CT methods.

### What Pedagogical Strategies Do Teachers Report Work Well for Using Computational Thinking Approaches in Teaching and Learning?

The data did not enable us to track and assess the outcomes of different pedagogic strategies. However, a rich variety of strategies was deployed by teachers, who also valued the opportunity to exchange practice with peers. This indicates that the pedagogic challenge is not to develop pedagogical strategies or resources for use by teachers, but rather to support them through peer exchanges and coaching.

### What Challenges Do Teachers Face During the Adoption of Computational Thinking in Classrooms?

The teachers unanimously identified challenges in the weaknesses in the technical infrastructure, large classes, lack of technical support and lack of resources. In the findings for research question 2, we saw that teachers did not need support in their technical skills, but they do need suitable equipment that is adequately maintained. All teachers also identified lack of instructional time as a major challenge. Mitigating strategies included the use of videos and adaptation of existing activities, support from colleagues and social media, and rotating groups of students from library activities to CT activities, and peer support and coaching would be valuable in helping teachers to meet the challenges they face.

#### Limitations

The limitations of this study are related to the population, which was drawn on a self-selecting sample of teachers who were involved in the MoE training course within which the research was carried out. This approach offered great advantages in access to teachers, but inevitably other teachers, who might have had valuable insights to offer, or who could have contrasted the results, were not included in the study. In addition, a smaller number of teachers were observed in their classrooms compared with the number of interviews, because not all interviewees volunteered to be observed. As a consequence of this self-selection of respondents, the research design adopted a qualitative approach.

#### Implications and Future Work

The study represents a first step toward understanding teachers’ experiences and students’ engagement in CT in Palestine. More research is necessary to evaluate the support that teachers need to understand the integration process, and the instructional strategies that could motivate different students to engage in CT. The current study offers valuable information for decision-makers in Palestine, and evidence which provides encouragement for further efforts to include more CT in K-12 settings in the Palestinian context. A repeated theme in these conclusions is the importance of exchanges with peers and coaching from both peers and experts. This is a strong indication of where resources would be best deployed in support of teachers in implementing CT methods in their teaching. However, it should be recognized that many of the challenges identified, particularly in our analysis of research question 4, are systemic in the educational environment in Palestine, and not limited to CT. These conditions inevitably act as a constraint on what can be achieved through peer exchanges and coaching.

In future research, it is planned to make use of a mixed methods approach, to involve more teachers in a larger sample, and to include students. The researchers do not differentiate in the data analysis between the results for different genders and teaching topics, but recognize that this is an important aspect to be investigated in future work. Finally, the fact that the researchers did not focus on a specific level of school, with the teachers coming from the three levels of K-12 education in Palestine (elementary, middle school, and high school). Further research should investigate integrating computational thinking into instruction at different levels. The study took place over 7 months of integrating CT into instruction, a process which changed over the duration of the study. In the future, it will be important to conduct research to understand how to create a sustainable culture of CT among schools and teachers and to identify the impact of using different learning strategies to engage students in CT concepts and skills in different curriculum areas.

## Data Availability Statement

The raw data supporting the conclusions of this article will be made available by the authors, without undue reservation.

## Author Contributions

DB: data analysis, discussion, and editing. DG: lead writer, methodology, and data validation. SA: design, data analysis, and conclusion. SS: writing and literature review. AG: data validation and discussion. FK: design, and methodology. ZK: writing and conclusion. All authors contributed to the article and approved the submitted version.

## Conflict of Interest

The authors declare that the research was conducted in the absence of any commercial or financial relationships that could be construed as a potential conflict of interest.

## Publisher’s Note

All claims expressed in this article are solely those of the authors and do not necessarily represent those of their affiliated organizations, or those of the publisher, the editors and the reviewers. Any product that may be evaluated in this article, or claim that may be made by its manufacturer, is not guaranteed or endorsed by the publisher.
